# Membrane remodeling by SARS-CoV-2 – double-enveloped viral replication

**DOI:** 10.12703/r/10-17

**Published:** 2021-02-22

**Authors:** Jagan Mohan, Thomas Wollert

**Affiliations:** 1 Membrane Biochemistry and Transport, Institut Pasteur, UMR3691 CNRS, F-75015, Paris, France

**Keywords:** SARS-CoV2, coronavirus, replication, autophagy

## Abstract

The ongoing pandemic of the new severe acute respiratory syndrome coronavirus (SARS-CoV-2) has caused more than one million deaths, overwhelmed many public health systems, and led to a worldwide economic recession. This has raised an unprecedented need to develop antiviral drugs and vaccines, which requires profound knowledge of the fundamental pathology of the virus, including its entry, replication, and release from host cells. The genome of coronaviruses comprises around 30 kb of positive single-stranded RNA, representing one of the largest RNA genomes of viruses. The 5′ part of the genome encodes a large polyprotein, PP1ab, which gives rise to 16 non-structural proteins (nsp1– nsp16). Two proteases encoded in nsp3 and nsp5 cleave the polyprotein into individual proteins. Most nsps belong to the viral replicase complex that promotes replication of the viral genome and translation of structural proteins by producing subgenomic mRNAs. The replicase complexes are found on double-membrane vesicles (DMVs) that contain viral double-stranded RNA. Expression of a small subset of viral proteins, including nsp3 and nsp4, is sufficient to induce formation of these DMVs in human cells, suggesting that both proteins deform host membranes into such structures. We will discuss the formation of DMVs and provide an overview of other membrane remodeling processes that are induced by coronaviruses.

## Introduction

Coronaviruses are enveloped viruses that belong to the order *Nidovirales*. They possess a positive-sense single-stranded RNA genome and are characterized by the production of subgenomic mRNAs during infection. The subfamily *Orthocoronavirinae* comprises alpha-, beta-, gamma-, and delta-coronaviruses that infect a broad range of amphibians, birds, and mammals. Alpha-coronaviruses comprise the human coronavirus NL63 that causes mild to moderate respiratory tract infections. Severe acute respiratory syndrome coronavirus 2 (SARS-CoV-2), together with SARS-CoV and MERS-CoV (Middle East respiratory syndrome–related coronavirus), belongs to the genus beta-coronavirus, which also comprises human coronavirus (HCoV)-OC43 and -HKU1. The latter cause mild upper respiratory tract infections such as the common cold, whereas MERS and SARS-CoV are responsible for severe forms of lower respiratory tract infections. The highest mortality rate was observed in patients infected with MERS-CoV (30%)^1^, followed by SARS-CoV^2^ (9%) and SARS-CoV-2 (<0.5%)^3^. However, the total number of deaths does not correlate with the mortality rate. For example, MERS-CoV and SARS-CoV-2 have caused 848 and 774 deaths, respectively, while SARS-CoV-2 has already killed more than 1,000,000 people^4–6^. The primary reason for the large number of Covid-19 cases and victims is related to the extremely rapid spread of the disease, which is triggered by a large proportion of asymptomatic infections and superspreading events^7–9^. Until an efficient treatment of the disease or a vaccine is found, social distancing and physical barriers are essential to control viral circulation and to limit the propagation of the virus.

Since the onset of the first coronavirus outbreak with SARS-CoV in 2002, our knowledge regarding the fundamental biology of coronaviruses has been tremendously advanced. However, many fundamental questions regarding the viral life cycle remain to be answered. In this review, we provide an overview of the molecular biology of SARS-CoV-2 and focus on membrane remodeling events that are induced by viral proteins to promote viral replication.

## Uptake of severe acute respiratory syndrome coronavirus 2: a plan B to avoid endocytic compartments

**Figure 1.**
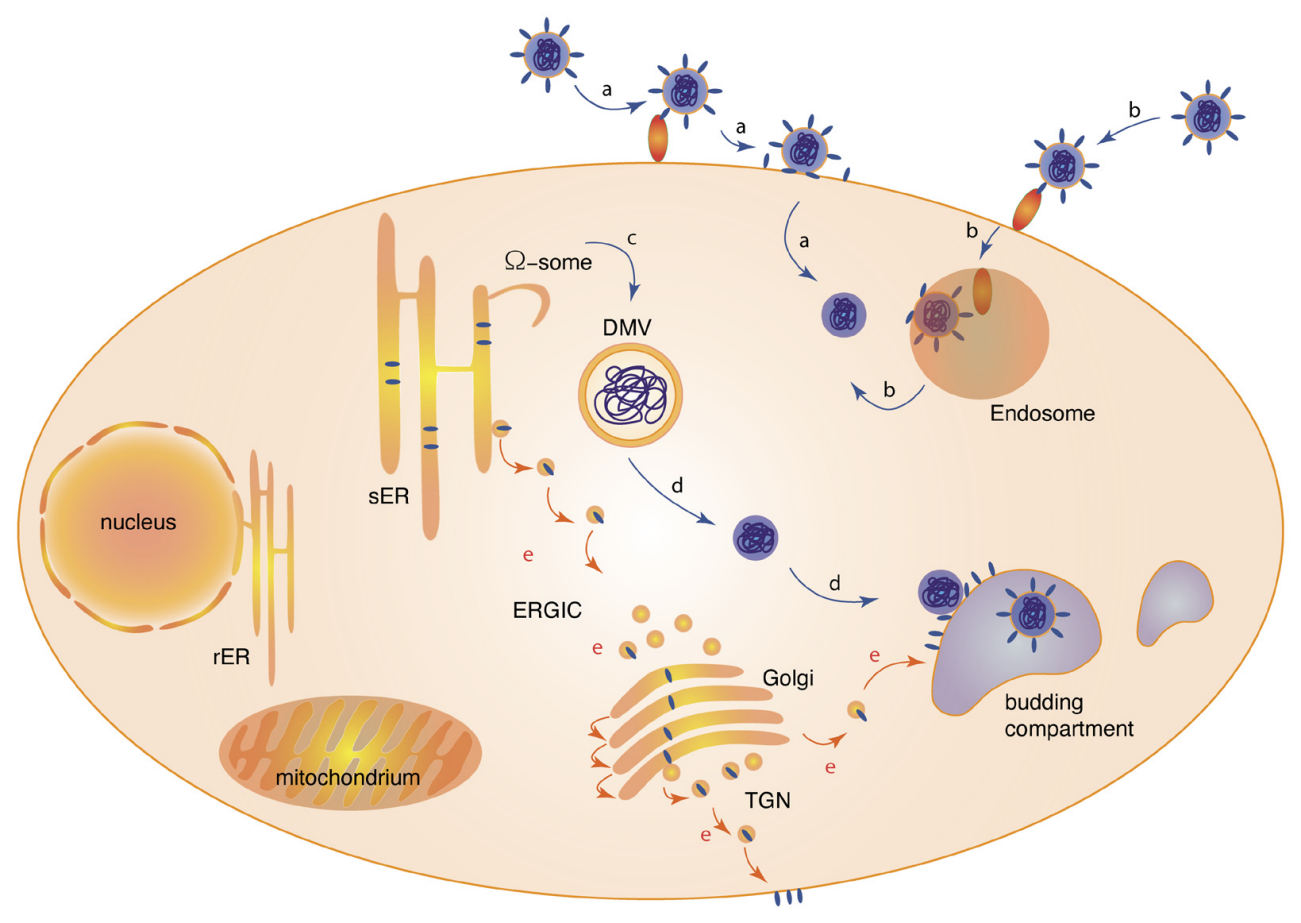
Infection cycle of severe acute respiratory syndrome coronavirus 2 (SARS-CoV-2). The infection cycle of SARS-CoV-2 is shown. (**a**) Viral entry by binding to the cell surface receptor angiotensin-converting enzyme 2 (ACE2) (red) and fusion of the spike protein after proteolytic processing at the plasma membrane by TMPRSS2. (**b**) Viral entry by taking advantage of the endocytic pathway involving clathrin-dependent or -independent endocytosis and proteolytic processing in lysogenic compartments involving cathepsin L. (**c**) Formation of double-membrane vesicles (DMVs) at the endoplasmic reticulum (ER). (**d**) DMVs serve as replication organelles to produce viral genomic RNA. The interaction of exported RNA and viral N-proteins gives rise to viral capsids (dark blue) that are delivered to budding compartments (light blue). Translation of structural proteins, including nucleocapsid (N), membrane (M), surface (S), and envelope (E) proteins, occurs from subgenomic mRNAs that are produced by the viral replication–translation complex. (**e**) Trafficking of viral structural proteins (dark blue) from the ER to the Golgi and the plasma membrane following the exocytotic pathway. Proteins accumulate in viral budding compartments that are derived from membranes of the exocytic pathway, where capsids bud into the lumen. The compartments fuse with the plasma membrane to release viruses. ERGIC, endoplasmic reticulum–Golgi intermediate compartment; rER, rough endoplasmic reticulum; sER, smooth endoplasmic reticulum; TGN, trans-Golgi network.

Coronaviruses are enveloped by a membrane derived from the host cell during budding. Three viral transmembrane proteins are embedded in this membrane: the spike (S) protein, the envelope (E) protein, and the membrane (M) protein. Much effort has been dedicated to characterize the S-protein of SARS-CoV-2 since this protein is key to enter cells and a primary target of the adaptive immune system of the host. The S-protein belongs to the well-characterized class I fusion proteins and shares high similarity with the S-protein of SARS-CoV, to hemagglutinin of influenza A viruses, and to the envelop protein of HIV^10^. The human receptor and binding partner for the S-proteins of both SARS coronaviruses is the angiotensin-converting enzyme 2 (ACE2)^11,12^. The firm interaction of the receptor binding (S1) domain of the S-protein with ACE2 promotes tight adhesion of the virus to the host cell plasma membrane^13^. Entry of viruses into the host cells depends on the fusion of viral envelope and host cell membranes (Figure 1a). The fusion process is triggered by the S2 domain of the S-protein, which harbors a fusion peptide. In order to be activated, the S-protein needs to be cleaved at two proteolytic cleavage sites. The first cleavage site of the SARS-CoV-2 S-protein, which is located between the S1 and S2 domains, is cleaved by furin-like proteases probably during S-protein biosynthesis^14^. The second cleavage site in the S2 domain is cut by the host cell protease TMPRSS2 after the S-protein has bound to ACE2 on target cells^11^. The S1 subunit is released and the S2 subunit undergoes a first conformational rearrangement to expose the fusion peptide. This is followed by an insertion of this peptide into the host cell membrane. A second large conformational change of the S2 subunit triggers fusion of the viral envelope and host cell membranes^15^. Since TMPRSS2 is a plasma membrane protein, cleavage and activation of the SARS-CoV-2 S-protein take place at the host cell plasma membrane. A similar mechanism has been reported for the uptake of SARS-CoV^16^. However, SARS-CoV and MERS-CoV can also be taken up into cells that do not express TMPRSS2 via clathrin-dependent endocytosis followed by cleavage and activation by the pH-sensitive protease cathepsin L (Figure 1b)^17–19^. Establishing the entry pathway of SARS-CoV-2 is of tremendous importance to understand its pathogenicity and to identify vulnerable cell types^20^. ACE2 and TMPRSS2 are highly enriched in nasal epithelial cells which correlates with viral replication in the upper respiratory tract^21^. The efficient colonization of these cells promotes viral spreading and infection and renders SARS-CoV-2 more contagious than other related coronaviruses^22^. Epithelial cells of the lower respiratory tract, including bronchial and small airway tissues, express significantly less ACE2 and TMPRSS2, suggesting that they are less susceptible to viral infection. However, clathrin-dependent and -independent endocytosis followed by the fusion of viral envelope and host endolysosomal membranes provides a second access route to host cells^23–25^. Lysosomotropic agents such as chloroquine inhibit entry of SARS-CoV-2, suggesting that this coronavirus can also be taken up by endocytosis, which contributes to the colonization of cells lacking TMPRSS2 (Figure 1b)^26^. Although the specific contribution of the two alternative routes for the entry of SARS-CoV-2 into host cells remains to be established, recent evidence suggests that clinical symptoms of patients with Covid-19 correlate with expression levels of ACE2 and TMPRSS2. In children, known to be less susceptible to SARS-CoV-2 infections, strongly reduced expression levels of ACE2 and TMPRSS2 in upper and lower respiratory tract tissues have been observed^27^.

## Critical functions but intrinsically disordered – viral non-structural proteins

**Figure 2.**
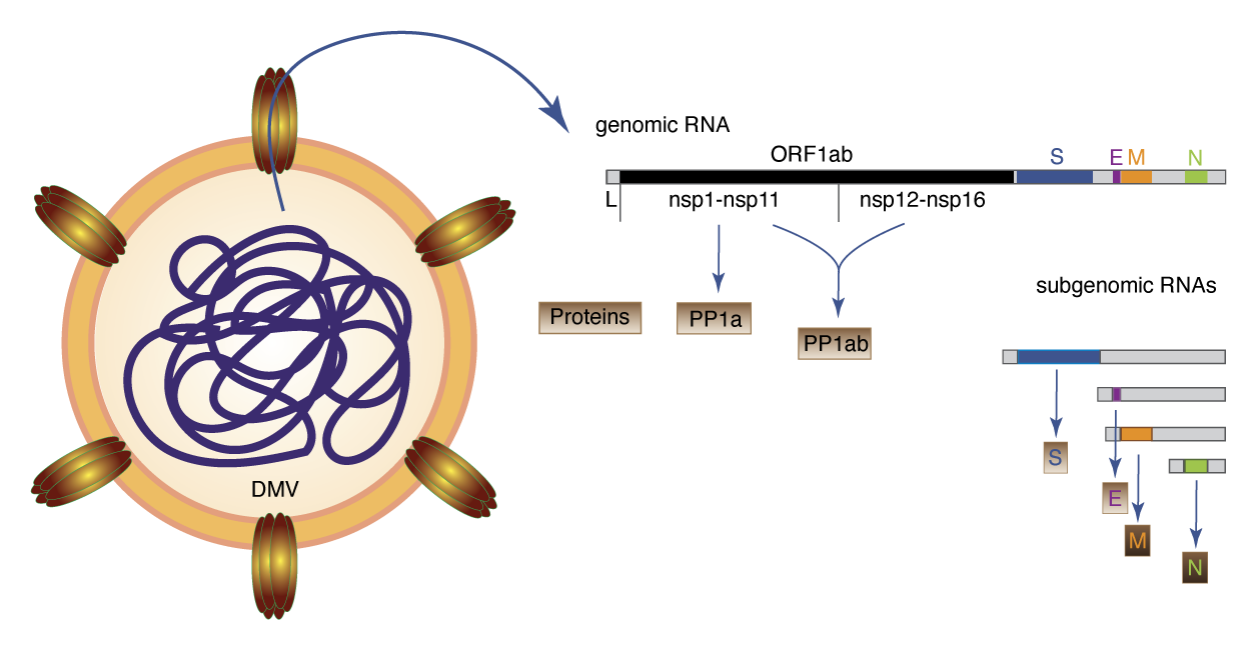
Replication and translation of viral genomic RNA. Double-membrane vesicles (DMVs) contain double-stranded viral RNA, which is an intermediate of viral genomic RNA replication. Channels that span the double membrane allow viral RNAs, including positive-strand viral genomic RNA and subgenomic RNAs, to be exported. Translation of open reading frame 1a (ORF1a) gives rise to the polyprotein 1a (PP1a) comprising nsp1 to nsp11. A programmed ribosomal frameshift leads to transcription of PP1ab comprising nsp1 to nsp16. Subgenomic RNAs contain a common 5′ leader sequence and their translation gives rise to S (surface), E (envelope), M (membrane), and N (nucleocapsid) structural proteins. The 3′ part of the genome comprises additional ORFs (3a, 6, 7a, 7b, 8, and 10) that are not depicted. Their transcription leads to additional subgenomic RNAs (not shown). L, leader sequence.

Whatever path is taken by viral particles to gain access into human cells, fusion of viral and host membranes releases viral capsids into the cytoplasm of host cells. The capsid contains the genome of CoVs, which is single-stranded positive-sense RNA that possesses a 5′ cap and a 3′ poly (A) tail. The genome thus serves as a template for translation. In the early phase of infection, the two partially overlapping open reading frames (ORFs) 1 and 2 of the genome are expressed which comprise two thirds of the total genome, giving rise to the two polyproteins (PPs) 1a and 1ab (Figure 2).

PP1a comprises 11 non-structural proteins (nsps) that are cleaved into single nsps by the papain-like protease nsp3 and the 3C-like protease nsp5. A programmed ribosomal -1 frameshift upstream of the stop codon of nsp11 allows continuation of translation and gives rise to PP1ab that is cleaved into 15 polypeptides, comprising nsp1 to nsp16^28^. Proteins that are generated by cleavage of PP1a modulate cellular pathways and remodel endomembranes of host cells to generate compartments for viral replication. Proteins, including nsp12 to nsp16, are derived from PP1b and, owing to the lower frequency of the programmed frameshift, are less abundant^29^. However, these proteins assemble into a complex that associates with host endomembranes to coordinate viral replication and translation reactions.

Nsp1 of SARS-CoV-2 shuts down translation of host mRNAs and RIG-I–dependent innate immunity by binding to the mRNA entry tunnel of ribosomes^30^. Nsp2 is dispensable for viral replication in cell culture and no specific function has yet been revealed^31^. The multidomain protein nsp3 combines various different functions^32^. Its papain-like protease (PL^pro^) cleaves nsp1, nsp2, and nsp3. Moreover, nsp3 is a major interaction hub and integral member of the viral replication complex. The macrodomains of nsp3 suppress the host immune response by exerting several enzymatic activities, including ADP-ribose-1“-phosphate phosphatase, de-mono-ADP-ribosylation, and de-poly-ADP-ribosylation^33–35^. Another important function of nsp3 in remodeling host cell endomembranes is related to its two transmembrane helices and requires its interaction with the transmembrane protein nsp4 and nsp6^36^. Moreover, nsp3 was recently shown to form part of a pore that spans double-membrane vesicles (DMVs)^37^. Such vesicles are generated from the endoplasmic reticulum (ER) and serve as viral replication organelles.

The two proteins nsp7 and nsp8 are co-factors of the main RNA-dependent RNA polymerase nsp12^38^ and their interaction enhances RNA binding and processivity of nsp12^39–41^. The two remaining proteins of PP1a—nsp9 and nsp10—are associated with the RNase complex through a direct interaction between nsp8 and nsp9^42^. They bind single-stranded RNA and are supposed to regulate or modulate the activity of nsp12 during the viral replication cycle^43^.

The C-terminal part of PP1a contains nsp11, which is a small polypeptide that becomes part of nsp12 upon programmed ribosomal frameshift. In addition to nsp12, PP1b contains proteins that are essential for viral replication, including the helicase nsp13 that unwinds RNA^44,45^. Replication of the large and complex viral genome of SARS-CoV-2 depends also on nsp14 that proofreads RNA during replication and corrects errors made by nsp12. The corresponding 3′-to-5′ exoribonuclease activity of nsp14 from HCoV-229E has been shown to be essential for the production of viable virus^46^. Nsp14 is also involved in mRNA cap formation by methylating the inverted guanosine moiety at the N7 position. The second methylation of the cap at the 2′-O position of the first transcribed nucleotide is added by nsp16^47^. Cap methylation also depends on nsp10, which interacts with nsp14 and nsp16, suggesting that cap methylating enzymes assemble together with the RNA polymerase and associated proteins nsp8, nsp9, and nsp13 to form part of a bigger replication complex^48^. Recent studies have started to reveal the molecular composition of the replication machinery, providing insights into the function of the complex^49–51^. Nsp15 is a nidoviral RNA uridylate-specific endoribonuclease (NendoU) which is involved mainly in suppressing antiviral immune responses by degrading cytoplasmic viral RNA^52,53^.

## Double is better than single – viral replication and double-membrane vesicles

Cells have evolved various antiviral strategies to counteract viral infection by detecting characteristic structures termed pathogen-associated molecular patterns (PAMPs). Double-stranded RNA (dsRNA) is a potent PAMP and is an intermediate that is produced during replication of RNA viruses, including SARS-CoV-2^54^. Pattern recognition receptors such as Toll-like receptor 3 as well as RIG-I–like receptors RIG-1 and MDA-5 detect dsRNA and activate signaling cascades that induce the production of type 1 interferons (IFNs), including IFN-α and IFN-β^55^. These cytokines induce antiviral responses in neighboring cells and activate innate and adaptive immune responses. Coronaviruses, including SARS-CoV and SARS-CoV-2, shield their dsRNA intermediates in DMVs, probably to evade IFN-1 activation^56^. Together with other strategies, including RNA capping and interference with antiviral signaling pathways, coronaviruses severely delay IFN-1 induction and corresponding pro-inflammatory immune responses^57,58^.

DMVs are abundant structures induced by many +RNA viruses, including arteriviruses^59,60^, hepatitis C virus^61^, noro- and picornaviruses^62,63^, and coronaviruses^64,65^. The morphology of these vesicles shows remarkable similarities with autophagosomes, and for some viruses, including SARS-CoV, a direct link between formation of DMVs and autophagy has been proposed^66^. Interestingly, expression of nsp3 and nsp4 appears to be sufficient to induce DMVs^67^. However, how these structures are formed remains elusive. Insights into potential molecular mechanisms for DMV formation come from transmission electron microscopy studies of cells that express single nsps or combinations of them.

Individually expressed nsp3 and nsp4 co-localize with ER markers, consistent with their co-translational insertion into the ER^67,68^. A recent study demonstrated that nsp3 forms together with other, yet-to-be-identified viral or host proteins pores that span DMVs, allowing for exchange of luminal and cytoplasmic material and for export of viral RNA^37^. The pore possesses a sixfold symmetry, and nsp3 is the major constituent of its cytoplasmic portion that possesses a crown-like structure. This suggests that the transmembrane domains of nsp3 span the cytoplasmic membrane of DMVs. Furthermore, nsp4 has been shown to interact with nsp3 and this interaction is required and sufficient to induce pairing of ER membranes^36^. Given that DMVs originate from the ER, it is tempting to speculate that nsp3 and nsp4 engage each other to form the pore with nsp4 spanning the inner membrane of DMVs. Consistent with this hypothesis, intraluminal loops of nsp3 and nsp4 are involved in the interaction of both proteins^36^. Whether DMVs directly emerge from these paired ER structures remains to be established.

A recent study provided evidence that DMVs are the primary site for viral RNA synthesis^56^. Moreover, electron-tomography of cells infected with the murine hepatitis coronavirus (MHV) revealed channel-like structures in DMVs, connecting the lumen of DMVs with the cytoplasm^37^. Although replication organelles of Flock House nodavirus (FHV) differ from DMVs, they also contain a pore opening toward the cytoplasm which shares remarkable structural similarity to channels observed in DMVs induced by MHV^69^. Moreover, this pore contains the FHV protein A, which harbors RNA polymerase and RNA capping activities, suggesting that these pores are central organization platforms for the viral replication and transcription machinery.

The major component of the protein pore in DMVs of MHV is nsp3, which lacks similar enzymatic activities. However, nsp3 of SARS-CoV was found to interact with nsp7 and nsp12, both of which form part of the viral replication and transcription complex^70^. Furthermore, nsp3 interacts with the N-protein, which together with viral RNA forms the capsid, suggesting that nsp3 is a central organization hub for viral replication and capsid assembly^71^.

## Double-membrane vesicles remain mysterious compartments

The generation of DMVs is not only a hallmark of cells infected by all coronaviruses. Similar structures are also observed in arteri-, noro-, and picorna-viruses and hepatitis C virus. Inhibiting their biogenesis can lead to potent antiviral therapies with a broad spectrum of action. This, however, requires that the molecular mechanism of DMV biogenesis is fully characterized and well understood. Unfortunately, our current knowledge is rather limited, and controversial observations regarding the biogenesis of DMVs have been made. This is particularly true for autophagy, one of the most versatile recycling pathways in eukaryotic cells.

Autophagy delivers damaged or superfluous cytoplasmic components, including organelles and protein aggregates, to lysosomes for degradation^72^. During this process, a double-membrane cup-like structure that engulfs autophagic cargo is formed. Sealing of this membrane gives rise to double-membrane limited autophagosomes. The obvious morphological similarity between such autophagosomes and viral DMVs suggests that viruses hijack autophagy-related (ATG) proteins to induce the formation of DMVs. Consistent with this hypothesis, ATG5, which is essential for canonical autophagy, was found to be required for MHV replication in embryonic stem cells^73^. By contrast, replication of MHV in mouse embryonic fibroblasts did not depend on ATG5^74^ or ATG7^66^, implying that the contribution of canonical autophagy to viral replication is cell type–specific. This, however, does not necessarily indicate that MHV replicates independently of autophagy in these cells because an ATG5/ATG7-independent autophagy pathway exists in embryonic tissues^75^.

Another potential connection between autophagy and viral replication involves LC3, an important autophagy protein and widely used cellular marker for autophagosomes. In canonical autophagy, LC3 is conjugated to the lipid phosphatidylethanolamine in autophagic membranes. The reaction is known as LC3 lipidation and involves conversion of LC3-I (non-lipidated) into LC3-II (lipidated) by a Ub-like conjugation system comprising E1 enzyme ATG7, the E2 enzyme ATG3, and the E3-ligase ATG12–ATG5-ATG16L1^76^. LC3 was found to co-localize with MHV nsp8, indicating that components of the autophagy pathways are indeed hijacked by MHV^73^. Moreover, depletion of LC3 strongly impaired MHV replication, suggesting that LC3 is required for viral replication^66^. Similar observations were made in SARS-CoV–infected cells^77^. By contrast, lipidation of LC3 was not required for replication of MHV or SARS-CoV in bone marrow–derived macrophages or mouse embryonic fibroblasts, although LC3 was found to co-localize with viral nsps^78^.

LC3-II tethers cargo to autophagic membranes through its interaction with autophagy receptors such as p62^79^. Given that DMVs are devoid of such cargo, it is plausible that LC3-II is dispensable for viral replication. This implies that nsps co-localize with LC3-I instead of LC3-II. Although autophagy-independent functions of LC3-II have been discovered in the past, the molecular function of LC3-I remained less well understood^80^. However, LC3-I appears to be involved in ER-associated degradation (ERAD), which is an ER stress response and quality control pathway that ensures that unfolded proteins are removed from the ER^81,82^. In this pathway, LC3-I was found to co-localize with vesicles that remove ERAD regulators from the ER. Thus, it is possible that coronaviruses coopt ERAD-related pathways by recruiting LC3-I to induce the formation of DMVs^81^.

Without any doubt, the ER plays a central role in the replication of coronaviruses. Many electron microscopy studies of infected cells demonstrated that ER membranes are heavily deformed and remodeled^56^. As discussed in the previous section, the viral proteins nsp3, nsp4, and nsp6 are central coordinators of such rearrangements. Nsp6 is of particular interest regarding the relationship between ER membranes, viral replication, and autophagy. Autophagosomes are formed at distinct domains of the ER which are enriched in phosphatidylinositol-3-phosphate (PtdIns3P) by the action of an autophagy-specific PtdIns3–kinase complex. PtdIns3P binding proteins, including DFCP1, are recruited to these domains and induce the formation of omegasomes, which are cradle-like extensions of the ER (Figure 1c). Omegasomes serve as platforms for autophagosome biogenesis by coordinating the recruitment of autophagy proteins and lipids^83,84^. Nsp6 of the avian coronavirus IBV (infectious bronchitis virus) was shown to co-localize with the PtdIns (3)P binding proteins DFCP1 and WIPI2 at the ER, suggesting that nsp8 initiates the formation of autophagosomes^85^. Similar observations have been made for nsp6 from MHV and SARS-CoV. However, expansion of such autophagosomes was limited by nsp6. As a result, much smaller autophagosomes were formed in nsp6-expressing cells^86^. Although expression of nsp6 is sufficient to induce autophagy, the formation of DMVs depends on the co-expression of nsp3 and nsp4. This suggests that nsp6 initiates the formation of omegasomes but that nsp3 and nsp4 are required to prevent formation of canonical autophagosomes by inducing the formation of DMVs (Figure 1c).

## Conclusions

Understanding membrane dynamics in cells is challenging. Even for fundamental transport processes between organelles, the biogenesis of organelles, and their degradation, many open questions remain. Revealing how complex viruses such as SARS-CoV-2 and other coronaviruses replicate and bud by manipulating cellular organelles and membrane trafficking pathways adds another layer of complexity. On the other hand, viral and bacterial pathogens have been excellent models to study fundamental cellular functions for a long time. Many insights into the dynamics of the actin cytoskeleton have been revealed by studying intracellular pathogens. Using coronavirus as models is a remarkable opportunity to reveal fundamental principles of membrane dynamics but also represents a challenge. This is particularly true for the budding process of coronaviruses, which is not at all understood at a molecular level^54^. Electron microscopy studies of cells infected with MHV revealed that virions bud into vacuolar structures that are derived from membranes of the secretory pathway, notably the ER–Golgi intermediate compartment and the Golgi^87^. However, these vacuoles possess remarkably different morphologies compared with canonical cellular organelles^88^. Furthermore, viral budding requires that viral RNA produced in DMVs and host membranes containing viral structural proteins converge in budding compartments (Figure 1e). This requires that structural proteins, including S- and M-protein of coronaviruses, are delivered to these structures. If the S-protein is expressed in human cells, it is transported by the canonical secretory pathway to the plasma membrane^89^. In infected cells, however, the S-protein needs to be diverted to budding compartments. How transport of viral capsid, donor membranes, and structural proteins is coordinated remains another open question and a major challenge for future research (Figure 1e).
